# Resistance induction with silicon in Hass avocado plants inoculated with *Phytophthora cinnamomi* Rands

**DOI:** 10.1080/15592324.2023.2178362

**Published:** 2023-02-22

**Authors:** Andree Álvarez, Diego Oliveros, Yalile C. Ávila, Angie Carolina Sabogal Palma, Walter Murillo, Jordi Eras Joli, María Bianney Bermúdez-Cardona, Nathalie Guarnizo

**Affiliations:** a Departamento de Química, Facultad de Ciencias, Universidad del Tolima, Ibagué, Colombia; b Instituto de Biología, Facultad de Ciencias Exactas y Naturales, Universidad de Antioquia, Medellín, Colombia; c Departamento de Química, Servicios Científico Técnicos-TCEM, Universidad de Lleida, Lleida, España; d Departamento de Producción y Sanidad Vegetal, Facultad Ingeniería Agronómica, Universidad del Tolima, Ibagué, Colombia; e Departamento de Química, ETSEA, Universidad de Lleida, Lleida, España

**Keywords:** Hass avocado, *Phytophthora cinnamomi*, silicon, defense induction, elicitor

## Abstract

Root rot caused by *Phytophthora cinnamomi* Rands, is one of the main factors that limits avocado production worldwide; silicon as a defense inducer seems to be a viable strategy to integrate into the management of this disease. Hereby, the present study evaluated the induction of resistance with silicon in Hass avocado plants inoculated with *P. cinnamomi*, as a possible alternative to conventional agrochemical management. A potassium silicate solution (10 mL, 0.2 M expressed as SiO_2_) was applied by irrigation, for ten days before inoculation with *P. cinnamomi* in Hass avocado plants. Leaf samples were taken at 3, 24, 144, and 312 h after inoculation with the pathogen. Peroxidase (POD) and polyphenol oxidase (PPO) enzymes had their highest activity 3 h after pathogen inoculation (p < .05). There was a decrease in the activity of the enzyme phenylalanine ammonialyase (PAL), in the content of total phenols, and the inhibition capacity of the DPPH^●^ radical, between 3 h and 24 h in the plants with the inducer and inoculated with *P. cinnamomi* (p < .05). The results suggest a beneficial effect of silicon as a defense inducer in Hass avocado plants, manifested in the activation of enzymatic pathways related to the regulation of oxidative stress and the synthesis of structural components. Therefore, the application of silicon as a defense inducer emerges as a strategy to include in the integrated management of the disease caused by *P. cinnamomi* in Hass avocado.

## Introduction

The Hass avocado is recognized for being one of the main Colombian export products, going from 1,760 tons exported in 2014 to 28,487 in 2017.^1^ Due to its favorable agroclimatic conditions, Colombia has reported the highest fruit yields (up to 9.85 t/ha in 2007).^2^ During 2021, a production of 214,618 tons was estimated, distributed in approximately 20,446 ha in Colombian territory.^3^ This increase in the national numbers for Hass avocado boosts optimization of the technological management packages, to maintain the competitiveness and sustainability of the crop.

In this aspect, one of the main factors that limit the production of avocado worldwide is root rot caused by *Phytophthora cinnamomi* Rands; This disease generates losses in commercial crops between 45 and 90%.^4^ In Colombia, between 30% and 50% of affected trees in the nursery stage have been reported, during the first two years of the establishment of the crop.^5^ The disease causes a progressive decline that eventually leads to the death of severely attacked trees. This is evidenced by a general wilting of the leaf area, also known as “sadness”.^2^ Initially, the plant presents mild to moderate partial defoliation and chlorosis, vegetative growth stops, and therefore, fruit production.^6^

Moreover, there is currently no consensus in the studies carried out for management against this pathogen. That is necessary to allow the design of effective control and prevention strategies for the disease, adding to the little knowledge about the plantpathogen interaction in avocado crops.^4^ The induction of resistance seems to be an effective alternative in attenuating the effect of the disease on avocado production.^7,8^

Hereby, different studies have focused on evaluating the role of silicon in plant/pathogen interactions, finding that its application can induce the structural reinforcement of the plant cell, the production of antimicrobial compounds, as well as increase the resistance of the plant. This response is associated with the activation of multiple signaling pathways and modulating the expression of defenserelated genes, which translates into stimulation of acquired systemic resistance.^9–11^

Additionally, the ability of silicon to induce a decrease in the incidence of fungal diseases and enhance resistance in monocots and dicots has been described.^12^ Fortuitously, the application of silicon in crops has been considered safe, since it requires a minimum concentration to attenuate the disease, can be as effective as a fungicide, and manages to increase partial resistance to almost the same level as complete genetic resistance.^12,13^ Therefore, this study evaluated the effect of silicon as an inducer of the defense response in Hass Avocado plants inoculated with *P. cinnamomi* Rands, by measuring enzyme systems associated with cell wall strengthening and phenolic compounds synthesis.

## 1. Materials and methods

### 1.1. Treatments

Six month old Hass avocado plants (plants grafted with Hass cup and Hass rootstock) were used. The plants were established under greenhouse conditions in individual pots (44 cm height and 22 cm diameter); 4 kg of soil was added. The treatments were based on the application of silicon and the inoculation with the pathogen (Table 1). For each treatment, different times were evaluated from the inoculation (Table 1). Three samples per treatment were arranged, each sample equivalent to two plants (six plants sampled in each treatment, for each time). The distribution of the plants in the greenhouse was random; Finally, the complete experiment was repeated 3 times in different periods: in November 2018, March 2019, and in April 2019; a total of 96 Hass avocado plants were used in each repetition, and a total of 288 in the entire experiment.

**Table 1.** Experimental design in each period. SiPc: Plants irrigated with Si and inoculated with *P. cinnamomi*; Si: plants irrigated with Si, without inoculating; Pc: plants inoculated with *P. cinnamomi* without Si; C: plants without application of Si and not inoculated. hpi: hours post inoculation.

Potassium silicate	*P. cinnamomi*	Treatment		Sampling time (hpi)			
				3	24	144	312
X	X	SiPc	Plants per repetition	6	6	6	6
X		Si		6	6	6	6
	X	Pc		6	6	6	6
		C		6	6	6	6

### 1.2. Silicon application

The application of silicon was done by direct irrigation in the soil for the SiPc and Si treatments. 10 mL of soluble potassium silicate, equivalent to 0.12 g of silicon dioxide (0.2 M SiO_2_), were applied daily for 10 consecutive days, for a total of 1.2 g of SiO_2_ per plant.^14,15^ On day 11, the plants of the corresponding treatments (SiPc and Pc) were inoculated with *P. cinnamomi*.^16^

### 1.3. *Inoculation of Hass avocado plants with* P. cinnamomi

The strain of *P. cinnamomi* used for the inoculation of the plants was supplied by the Corporation for Biological Research (CIB); the microorganism was replicated two weeks before in a culture medium for fungi, PDA (potato dextrose agar), taking discs of mycelium from the original culture. A transverse cut (1 cm) was made in the stem of the Hass avocado plant with sterile blades, 5 cm above the grafting point; Subsequently, an agar disc with mycelium of the pathogen with a diameter of 5 mm was inserted into the plant wound and the wound was sealed with Parafilm.^16^

### 1.4. Sampling for biochemical analysis

Leaves of the different treatments were taken at the corresponding times. Sampling was carried out between 10:00 a.m. and 1:00 p.m. at the maximum photosynthetic efficiency of the plant.^17^ The sampled leaves were immediately frozen with liquid nitrogen; the plant material was placed in labeled aluminum foil bags and stored at −80°C. Subsequently, the plant material was crushed using ceramic mortars with liquid nitrogen to maintain the biochemical integrity of the material. The leaves were stored in 50 mL conical tubes at −80°C until used for biochemical tests.^18^

### 1.5. Pigment quantification

The determination of the content of chlorophylls a, b, and carotenoids was carried out based on what was reported by^19^, from an 80% acetone extract of the tissue sampled in each treatment.

### 1.6. Quantification of phenols and inhibition of the DPPH^●^ radical

The Hass avocado leaf extract for the quantification of phenols was obtained with acetone (60%) to which the quantification of phenolic compounds was carried out by the Folin-Ciocalteu method.^20^ From the same extract, the inhibition capacity of the 1,1-diphenyl-2-picrylhydrazyl [DPPH^•^) radical was measured, using the method reported by^21^,with some modifications: Trolox was used as a standard at concentrations between 4.99 and 79.99 µM and was evaluated against the radical; DPPH^•^ absorbance values were measured at 517 nm after 10 minutes of reaction using a 96-well UV/VIS microplate reader (Multiskan® GO Thermo scientific]; each reaction consisted of 200 µL of the radical and 50 µL of the extract to be evaluated.

### 1.7. Phenylalanine ammonia-lyase [PAL) activity

The measurement of the PAL activity of the samples obtained was determined based on^22^, with some modifications: extraction buffer (50 mM borate buffer, pH 8.8] and reaction buffer (50 mM borate buffer + 20 mM Phenylalanine, pH 8.8) were prepared. In a 1:20 ratio, the extraction buffer was applied to the sample. It was centrifuged at 11,000 rpm, 4°C, and 10 min. The supernatant (enzymatic extract) was taken. 200 μL of reaction buffer was added to 20 µL of enzyme extract. The blank consisted of 20 µL of extraction buffer and 200 µL of reaction buffer. After 4 min of reaction, it was read at 290 nm in a microplate reader every 10 seconds for 10 min to obtain kinetics of PAL enzyme activity. This was done at a constant temperature of 37°C. One unit of PAL activity is defined as the amount of enzyme that generates an increase in absorbance of 0.01 at 290 nm h^−1^. The enzyme activity was expressed as units of enzyme activity per mg of protein (U*mg^−1^).

### 1.8. Peroxidase [POD) activity

The method to evaluate POD activity followed the procedure based on what was reported by^23^, with some modifications: extraction buffer was prepared (100 mM Na-phosphate buffer, pH 7.0], and reaction buffer or substrate (100 mM Na-phosphate buffer, 20 mM guaiacol, pH 7.0). In a 1:20 ratio, the extraction buffer was applied to the sample and homogenized. Subsequently, they were centrifuged at 11,000 rpm, 4°C, and 30 min. The supernatant (enzymatic extract) was taken. 144 µL of reaction buffer was applied to 36 µL of enzyme extract. They were incubated for 5 min at 30°C. 72 µL of H_2_O_2_ (100 mM) was applied to the mixture and the absorbance at 460 nm for 2 min was measured. The specific activity of the enzyme was calculated as ∆Abs 460 min^−1^mg protein^−1^ and expressed as units of enzyme activity per mg protein (U*mg^−1^).

### 1.9. Polyphenol oxidase (PPO) activity

The extraction buffer (100 mM Na-phosphate buffer, pH 7.0) and the reaction buffer (0.1 M Na-phosphate buffer, 0.1 M catechol, pH 7.4) were prepared. In a 1:20 ratio, the extraction buffer was applied to the sample. Subsequently, it was centrifuged at 11,000 rpm, 4°C, and 20 min. The supernatant (enzyme extract) was taken. 3 mL of reaction buffer or substrate was applied to 100 µL of enzyme extract. The oxidation rate of catechol was monitored at 410 nm, at 25°C for 1 min. PPO activity was calculated as the change in optical density unit (410 nm) g^−1^ FW min^−124^ and expressed as units of enzymatic activity per mg of protein [U*mg^−1^ of protein)

### 1.10. Protein quantification of enzyme extracts

Protein quantification in enzyme extracts was determined by the method of^25^,with appropriate modifications at a protein concentration between 100 and 1 µg/mL^26^.

### 1.11. Statistical analysis

The reported results correspond to the mean of nine determinations ± standard deviation. A one-way analysis of variance (ANOVA) was performed with a significance level of 0.05, to which the assumptions were verified [Levene’s test confirms the assumption of homogeneity between treatments (p > .01) and the Shapiro-Wilk test confirms that the data show a normal distribution (p > .05)]. Additionally, a comparison of means was performed using the Tukey test.

## 2. Results

### 2.1. *Silicon pre-treated plants had fewer symptoms after being inoculated with* P. cinnamomi

The irrigation with potassium silicate was directly on the moistened soil of each Hass avocado plant, for 10 consecutive days with a 24-h difference between each application, until inoculation with the pathogen on day 11. The application of silicon was carried out previously to allow the assimilation of the nutrient via the root, corresponding to what was observed by different authors during similar tests of defense induction with silicon in commercial species.^27–29^ Visually, the plants to which silicon was supplied did not show any difference from the others at the end of 10 days.

**Figure 1.**
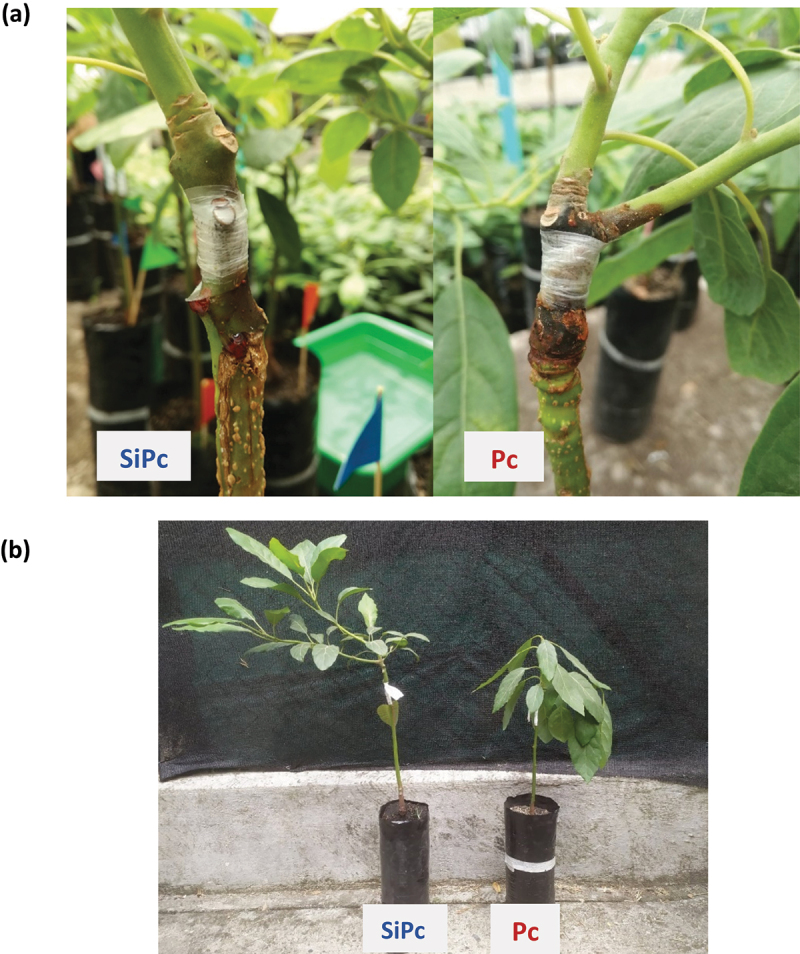
Comparison of the state of the plants in the SiPc and Pc treatments, 312 hpi. (a) The appearance of the lesion in the inoculation zone of *P. cinnamomi*. (b) Fitness of plants inoculated with *P. cinnamomi* with and without silicon treatment. SiPc: plants irrigated with Si and inoculated with *P. cinnamomi*; Pc: plants inoculated with *P. cinnamomi* without Si.

The symptomatology of the inoculated plants was notable for the darkening of the wound area, the tissue around it becoming necrotic, followed by a radial growth of the necrosis along the stem, with a mainly apical orientation (Figure 1a); Subsequently, necrosis invaded the space of the branches and a generalized wilting of the plant with the loss of tonicity was manifested, despite having an adequate water supply (Figure 1b). Those leaves and plants that showed accelerated wilting over the others, with signs of external conditions resulting from mechanical damage or possible herbivory, were not considered in the sampling carried out for the biochemical analyses.

**Figure 2.**
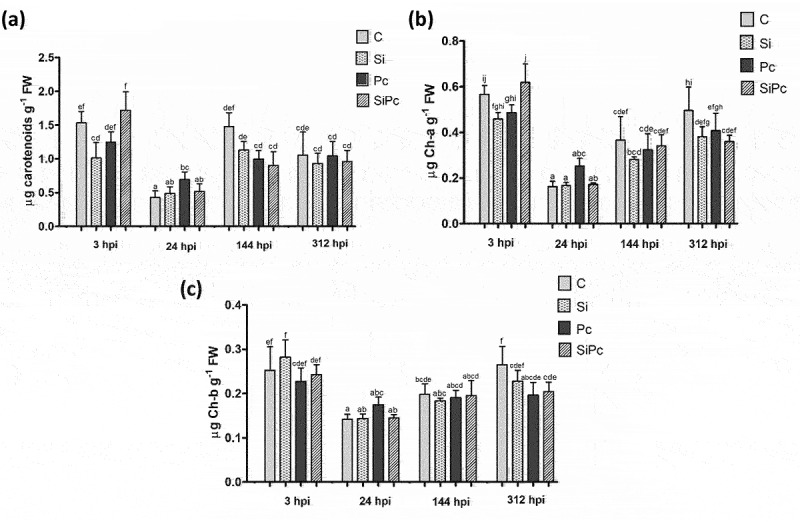
The total content of pigments in each treatment over time. a) Chlorophyll a content. b) Chlorophyll content b. c) Content of carotenoids. Ch-a: chlorophyll a; Ch-b: chlorophyll b; FW: fresh weight. hpi: hours post inoculation with *P. cinnamomi*. C: plants without potassium silicate application without *P. cinnamomi* inoculation; Si: plants irrigated with potassium silicate, without *P. cinnamomi* inoculation; Pc: plants inoculated with *P. cinnamomi* and without potassium silicate; SiPc: plants irrigated with potassium silicate and inoculated with *P. cinnamomi*. Data are presented as the mean ± standard deviation of nine replicates. The mean with the same letter is not significantly different (ANOVA followed by Tukey’s test with p < .05).

Chlorophyll content showed a significant decrease in the Pc treatment, from SiPc after 3 hpi; however, there were no significant changes between treatments in the content of chlorophylls a, b, and carotenoids (Figure 2), in the other times evaluated. These results suggest, that under the evaluated conditions, there was no affectation of the photosystems in the Hass avocado plants, even in those plants that presented a greater decay, as in the case of those subjected to the Pc 312 hpi treatment (Figure 1b).

### 2.2. *The presence of* P. cinnamomi *induces a higher content of phenolic compounds in the first hours of inoculation*

For the quantification of total phenols, the leaf samples were kept frozen at −80°C from the moment of collection until obtaining the extracts, to avoid the action of enzymes such as polyphenol oxidases, which could degrade these components of interest.^30^ The quantification method used sought to know the total phenolic compounds, however, the type of phenols found in the extracts or the proportion of these, against other secondary metabolites was not discriminated. Meanwhile, it was wanted to know the radical stabilization capacity of these phenolic compounds, so the DPPH^●^ anion stabilization test was carried out, a chromophore agent that decreases its absorbance at 517 nm when it is reduced by an antioxidant either by electron transfer or by giving it protons (H^+^).^31^

**Figure 3.**
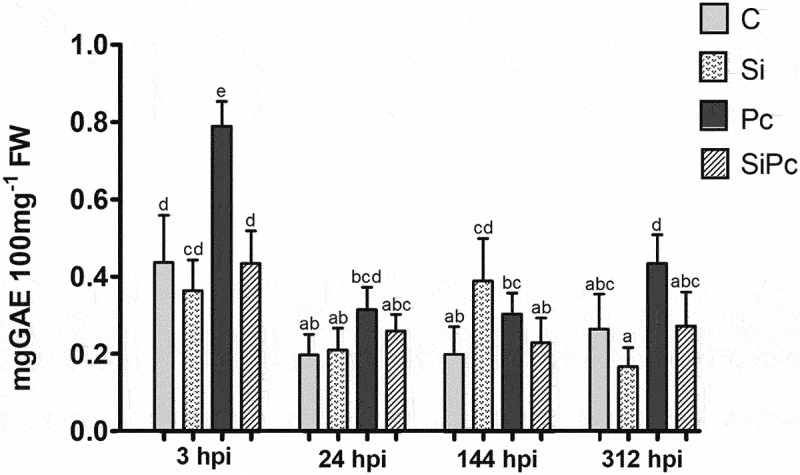
Total phenol content per treatment over time. GAE: gallic acid equivalents. FW: fresh weight. hpi: hours post inoculation with P. cinnamomi. C: plants without potassium silicate application without P. cinnamomi inoculation; Si: plants irrigated with potassium silicate, without P. cinnamomi inoculation; Pc: plants inoculated with P. cinnamomi and without potassium silicate; SiPc: plants irrigated with potassium silicate and inoculated with P. cinnamomi. Data are presented as the mean ± standard deviation of nine replicates. The mean with the same letter is not significantly different (ANOVA followed by Tukey’s test with p < .05).

**Figure 4.**
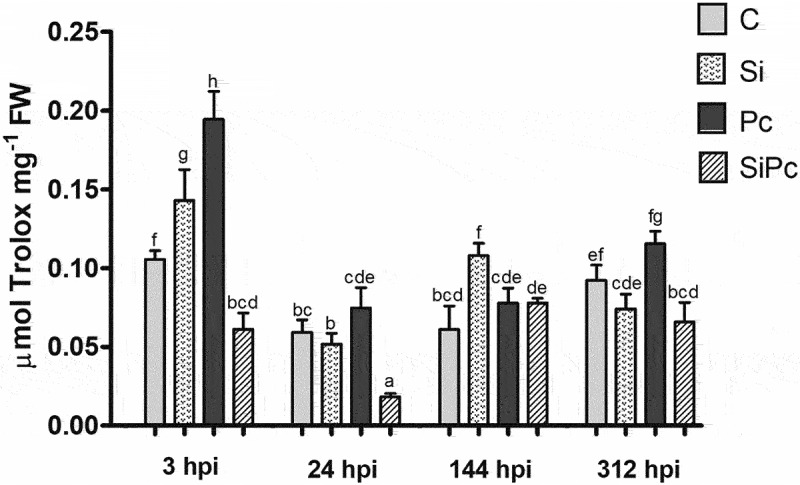
Stabilization capacity of the DPPH^●^ radical over time. FW: fresh weight. hpi: hours post inoculation with P. cinnamomi. C: plants without potassium silicate application without P. cinnamomi inoculation; Si: plants irrigated with potassium silicate, without P. cinnamomi inoculation; Pc: plants inoculated with P. cinnamomi and without potassium silicate; SiPc: plants irrigated with potassium silicate and inoculated with P. cinnamomi. Data are presented as the mean ± standard deviation of nine replicates. The mean with the same letter is not significantly different (ANOVA followed by Tukey’s test with p < .05).

Precisely, Figure 3 shows that the Pc treatment showed the highest content of phenolic compounds at 3 hpi, differing significantly from the others. This seems to coincide with what is shown in the stabilization capacity of the radical DPPH^●^ (Figure 4). These data suggest that during this period, there is a marked metabolic activity that could be involved in the defensive response of Hass avocado plants. It has been shown that the increase in phenolic compounds is a response mechanism in the presence of mycelium.^32^

The stabilization capacity of DPPH^●^ shows a marked decrease up to 24 hpi in all treatments. Just as the Pc treatment showed the highest content of phenols at 3 hpi, it also showed the most abrupt decrease in the concentration of phenols and antiradical activity, in the period between 3 h and 24 h; Taking into account that Hass avocado is susceptible to the disease caused by *P. cinnamomi*, it can be thought that this decrease in the concentration of phenols in plants without the presence of silicon as an inducer implies a decrease in the ability of the host to regulate ROS and RNS via these metabolites. From 24 hpi, the variations in the phenol content and antiradical activity of the Pc treatment are less marked; It should be noted that in the stem of each inoculated plant the growth of the necrotic lesion of the tissue surrounding the inoculation wound was maintained (Figure 1).

### 2.3. PAL, POD, and PPO enzyme activities, early defense response

**Figure 5.**
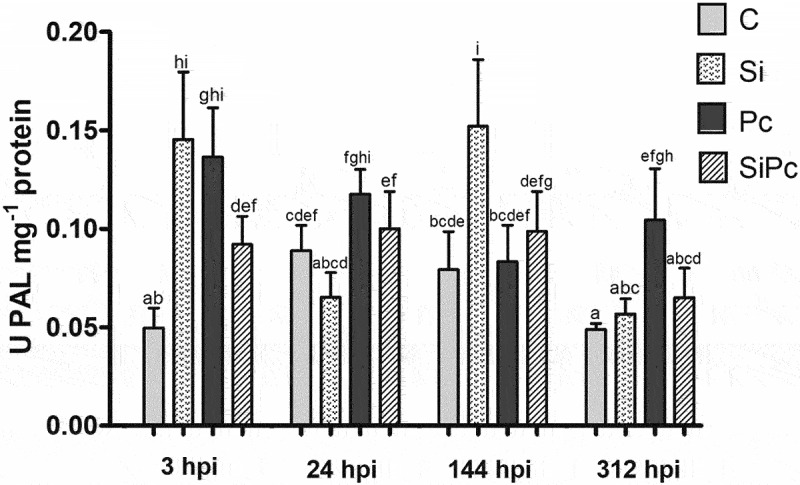
PAL activity per treatment over time. U: enzyme activity units. hpi: hours post inoculation with P. cinnamomi. C: plants without potassium silicate application without P. cinnamomi inoculation; Si: plants irrigated with potassium silicate, without P. cinnamomi inoculation; Pc: plants inoculated with P. cinnamomi and without potassium silicate; SiPc: plants irrigated with potassium silicate and inoculated with P. cinnamomi. Data are presented as the mean ± standard deviation of nine replicates. The mean with the same letter is not significantly different (ANOVA followed by Tukey’s test with p < .05).

**Figure 6.**
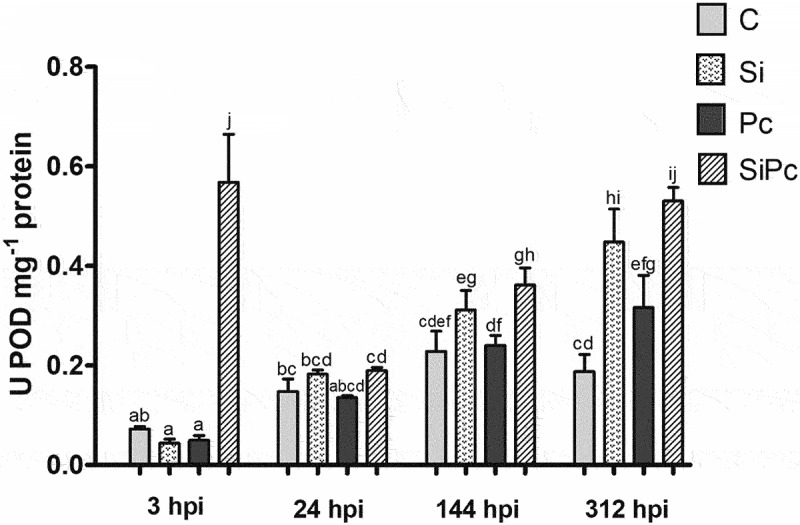
POD activity per treatment over time. U: units of enzyme activity. hpi: hours post inoculation with *P. cinnamomi*. C: plants without potassium silicate application without *P. cinnamomi* inoculation; Si: plants irrigated with potassium silicate, without *P. cinnamomi* inoculation; Pc: plants inoculated with *P. cinnamomi* and without potassium silicate; SiPc: plants irrigated with potassium silicate and inoculated *with P. cinnamomi*. Data are presented as the mean ± standard deviation of nine replicates. The mean with the same letter is not significantly different (ANOVA followed by Tukey’s test with p < .05).

**Figure 7.**
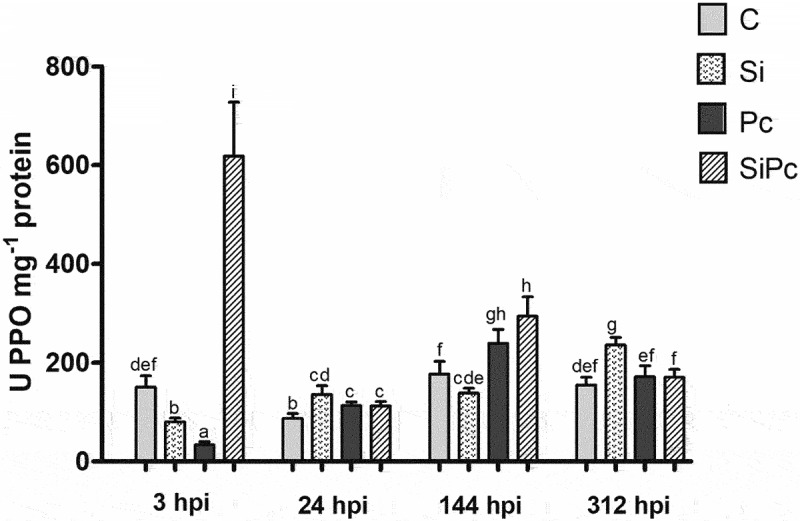
PPO activity per treatment over time. U: units of enzyme activity. hpi: hours post inoculation with *P. cinnamomi*. C: plants without potassium silicate application without *P. cinnamomi* inoculation; Si: plants irrigated with potassium silicate, without *P. cinnamomi* inoculation; Pc: plants inoculated with *P. cinnamomi* and without potassium silicate; SiPc: plants irrigated with potassium silicate and inoculated *with P. cinnamomi*. Data are presented as the mean ± standard deviation of nine replicates. The mean with the same letter is not significantly different (ANOVA followed by Tukey’s test with p < .05).

From the vegetal material of Hass avocado plants, the enzymatic extracts corresponding to the PAL, POD, and PPO enzymes were obtained. Activities were calculated based on enzyme kinetics and expressed as U*mg-1 of protein. Figures 5, 6, and 7 show the behavior over time of the PAL, POD, and PPO enzymatic activities, respectively.

Regarding the enzymatic activity of PAL, the application of silicon in Hass avocado plants does not seem to induce an increase in the presence of *P. cinnamomi* (Figure 5), this is contrary to what has been reported in different crops of commercial interest against P. their respective pathogen, after the application of silicon as a defense inducer.^33–35^ This situation seems to coincide with what was evidenced in this study in the content of phenols (Figure 3), where instead of increasing the content of these metabolites, it decreased in the SiPc treatment.

Concerning the other treatments, at 3 hpi, treatment C presented the lowest PAL activity of the entire experiment, with statistically significant differences compared to the other treatments. At the same time, the SiPc treatment was the closest to the activity of the plants without infection and did not present significant changes in the activity of this enzyme over time. Treatment C presented a similar behavior, adding to a marked increase in the activity of the PAL enzyme up to 24 hpi.

In Figure 6, it is possible to show the behavior in the different treatments over time of the POD enzymatic activity. As mentioned, at 3 hpi of the SiPc treatment, the highest activity of this enzyme was present, for an abrupt decrease in activity in said treatment up to 24 hpi; It is from this last time that the behavior in the different treatments is similar, with an increase in enzymatic activity until 312 hpi, unlike treatment C, which between 144 and 312 hpi does not present significant changes.

At 24 hpi, it cannot be affirmed that there are significant differences between the 4 treatments studied. Treatment C did not show significant changes over time; this was the only treatment that presented such behavior.

The behavior of the PPO enzyme evidenced in Figure 7 is like that described for POD, its activity being inversely proportional to the content of phenolic compounds of the SiPc and Pc treatments at 3 hpi. This situation is natural, considering that PPO is the main enzyme in the oxidation of phenols;^36^ PPO activity has been positively correlated with resistance to diseases induced by silicon, thanks to its participation in the synthesis of lignin and the increase in the antimicrobial capacity of host plants.^37^

The PPO activity also makes it possible to show again that the greatest differentiation between the treatments occurs only 3 hpi after the inoculation of the pathogen. At this time, each treatment presented significant differences from the others; This is perhaps because the main metabolic changes generated in the plant defense response occur in the first hours, emphasizing that for future research it is necessary to evaluate the period between 0–24 hpi.

After 24 hpi, the treatments with the presence of the pathogen behaved similarly, with an increase in PPO activity up to 144 hpi, followed by a decrease in it up to 312 h. On the other hand, the plants treated with silicon, without the presence of the pathogen, did not show abrupt changes in their enzymatic activity.

## 3. Discussion

### 3.1. Pigments content suggests similar oxidative stress levels between treatments

Chlorophyll a and b levels tend to change under conditions of high oxidative stress in plants, as their synthesis and accumulation are inhibited.^38^ The low variation in pigments content, between treatments over time, suggests that the oxidative conditions were not contrasting beyond 3 hpi, in the presence of the pathogen and the inducer in the Hass avocado plants.

From the above, only at the time of 3 hpi was there a significant difference in the content of chlorophyll a between SiPc and Pc. In this sense, it has been reported that silicylated plants tend to increase the activity of enzymes related to the protection of photosystems, such as superoxide dismutase (SOD), ascorbate peroxidase (APX), and peroxidases (POD), increasing in consequently the content of chlorophylls.^39^ This coincides with what was evidenced in the SiPc treatment at 3 hpi, where the POD activity was significantly higher than that of the other treatments (Figure 6).

The inoculation with *P. cinnamomi* in the Pc treatment showed more noticeable symptoms (necrosis and loss of turgor) compared to that evidenced in the SiPc treatment at 312 hpi (Figure 1b). The symptoms shown have been associated with the disease generated by the oomycete under study.^40,41^ Despite this effect on plant fitness, photosynthetic pigments and carotenoid content did not show significant differences between treatments inoculated, and with or without silicon at 312 hpi. Therefore, it could be thought that the loss of turgor observed is a consequence of parameters not evaluated, unrelated to oxidative stress.

Despite the above, changes in chlorophyll content are a suggested parameter for the detection and monitoring of diseases caused by pathogens.^42^ Similarly, changes in pigment content have been associated with abiotic stress in plants (mainly salinity stress).^39,43,44^

### 1.1. Phenolic compounds would be precursors of physical barriers and bioactive metabolites during the defense response of Hass avocado

The non-enzymatic mechanisms for regulating the oxidative processes that occur in the plant defense response involve phenols.^45^ However, the role of phenolic compounds in the plant defense response is not always related to the regulation of ROS and RNS.^46,47^ These compounds would fulfill different roles: structural and cell wall strengthening compounds,^48,49^ phytoalexins, phytoanticipins, antimicrobial compounds, pathogenicity modulators, defense gene activators,^50^ and also as radical stabilizing agents.^45^

The regulation of ROS and RNS is essential in the plant defense response, since these substances can become toxic to the plant, leading to cell death,^51^ however, this can be desired under certain conditions to prevent the advancement of biotrophic pathogens during the hypersensitive response (HR);^52^ In addition, ROS and RNS can trigger intracellular and intercellular signaling behind the systemic host response,^53^ or act as antimicrobial compounds [Muhammad]^54^.

The results of the Pc treatment coincide with the report by^55^, where the content of phenols in avocado rootstocks increases in the presence of *P. cinnamomi*. Thereby, it has been found that in roots of avocado trees infected with the same pathogen, and subjected to previous treatment of silicon dioxide, the concentration of these metabolites also increased, suggesting that upon contact with the pathogen they function as physical barriers, conferring a certain resistance to the penetration of *P. cinnamomi* to the cell wall.^14^

One of the characteristics of the defense responses based on phenols is the rapid and early accumulation in the infection zones, isolating the pathogen at the original site of entry to the host.^55^ This would explain the increase in these compounds at 3 hpi in the presence of *P. cinnamomi*. The SiPc treatment had a behavior oriented mainly to the decrease in the concentration of phenols and the radical stabilization capacity, suggesting that the defense response against the pathogen in silicylated Hass avocado plants, is not directly dependent on the biological activity derived from phenolic compounds.

Concerning total phenols, the SiPc and C treatments show similar behaviors during the first hours of the experiment It can be inferred that despite the presence of the pathogen *P. cinnamomi*, SiPc behaves in the same way as a not inoculated plant, thus avoiding the metabolic wear that entails the *de novo* synthesis of these metabolites.^56^

In avocado crops, potassium silicate has been used to control fruit diseases, in addition to root rot, varying the mode of application between foliar, root, and stem injection; among these, the foliar application has not shown an apparent effect on the defense response.^40,57,58^ The root application of silicon is more effective for the induction of defense in avocado; likewise, an increase in soluble phenolic compounds has been reported due to the root application of potassium silicate, being mainly glycosylated phenols, further suggesting that the undetected phenols are due to this fact as they are not water-soluble and are bound to the cell wall.^14,59^

It could be assumed that the decrease in the content of phenolic compounds in the SiPc treatment was due to the polymerization of such compounds in structures for strengthening the cell wall, or due to the oxidation of these metabolites. The production and transformation of soluble phenols are regulated by defense enzymes such as PAL, POD, and PPO.^33^ Both, the polymerization, and the oxidation of phenols would explain the decrease in antiradical activity.

### 1.1. The activity of the enzymes PAL, POD and PPO respond to the presence of the pathogen before that of Silicon, explaining the changes in the content of phenols

It has been shown that silicon can stimulate the activity of enzymes related to disease resistance during plant-pathogen interaction.^60,61^ Among these enzymes are phenylalanine ammonium lyase (PAL), peroxidases (PODs), and polyphenol oxidases (PPOs), which influence the content of phenolic compounds.^11^

This is how PAL catalyzes the deamination of the amino acid L-phenylalanine, generating trans-cinnamic acid as a product, which is the precursor of multiple types of phenols in the phenylpropanoid pathway, with lignin as the final product.^62^ On the other hand, cell wall stiffness is, in most cases, the result of POD-mediated H_2_O_2_-dependent crosslinking and its participation in the final steps of lignin biosynthesis from phenolic compounds.^11,63^ While PPOs are enzymes that catalyze the oxidation of monophenols and o-diphenols to o-quinones; the latter being highly reactive, generating secondary reaction products that include potentially cytotoxic ROS and o-quinone protein complexes, which generate the browning commonly observed in a wound on fruits.^64^

The increase in PAL activity is typical behavior against stress generated by cuts, such as that performed for the inoculation of *P. cinnamomi*, in a defensive response to strengthen the cell wall around a wound (SiPc treatments and PC).^65,66^ The PAL enzyme increased its activity in the Pc treatment but not in SiPc at 3 hpi; it is known that the expression of genes that code for phenylalanine ammonia-lyase (PALa and PALb), can be regulated with silicon treatments.^67^ This decrease in PAL activity in SiPc could be due to a preexisting systemic strengthening in the tissue of Hass avocado plants due to the accumulation of silicon.^11,58,59,68^ Regarding this,^69^,proposed a hypothesis that silicon deposition in the plant apoplast can interfere with pathogen effectors, preventing the pathogen from inhibiting the plant defense response [Jie]^70^.

The Si treatment showed one of the most erratic behaviors through the different times evaluated for the PAL activity; comportment was associated with the changes seen in the content of phenols. The main differences between treatments in the activity of the PAL enzyme are evident before 24 hpi. It is recommended for future research to evaluate the behavior of PAL activity in Hass avocado at multiple points throughout the period between 0 and 24 hpi.

It has also been shown that the activity of the PAL enzyme tends to increase through elicitation with salicylic acid (SA),^71^ while it is through PAL that the biosynthetic pathway of the same phytohormone occurs.^72^ This allows us to infer that the SA signaling pathway did not have significant activity in Hass avocado plants inoculated with *P. cinnamomi* and treated with silicon (SiPc). At the same time, SA can also negatively interfere with POD-mediated metabolic pathways; SA-induced systemic acquired resistance (SAR) is normally mediated by elevated ROS levels, achieved through inhibition of enzymes such as catalase and POD.^63^ Silicon may then have enhanced signaling by jasmonic acid (JA) in the evaluated treatments.

Regarding the regulation of POD expression, it is known that there is a joint action between SA and JA, however, a large number of POD isoforms can be induced by SA,^73,74^ but not in a generalized way, since some PODs do not respond to this induction.^63,75^ For its part, JA and its derivative, methyl jasmonate (MeJA), have been proposed as key compounds in the positive regulation of POD enzyme expression during its participation in plant defense responses, serving as a factor of transcription of Prx genes (genes encoding enzymes with peroxidase activity) [Mohammad^76–78^]. This information reinforces the approach that the main signaling pathway in the interaction between silicylated avocado plants and *P. cinnamomi* is given by the JA.

The POD would allow a strengthening of the cell wall of the Hass avocado plants, as it is an oxide-reducing enzyme with participation in the suberization of cellulose and the oxidation of phenols for the lignification of the cells in the defense response.^79^ Both, lignification and suberization, involve the formation of a three-dimensional polyphenolic matrix within the carbohydrate matrix of the primary cell wall,^80^ making these compounds neither soluble nor available for quantification with the Folin-Ciocalteu method used in the present study. This phenolic component is distinguished by the presence mainly of p-hydroxycinnamic, p-coumaric, caffeic, and ferulic acids, which constitute the cell wall-bound polyphenolic domain (PPD).^81^ A more detailed study, of the composition of phenols present in the evaluated samples, would allow the presence of the mentioned phenolic acids to be evidenced.

It has been reported that POD activity increases with the administration of silicon, in plants without pathogen.^82^ This is contrary to what was found in this investigation, where the trend at each time was the highest POD activity in the SiPc treatment (Figure 6). In this same sense, the decrease in the content of soluble phenols between 3 and 24 hpi of the SiPc treatment (Figure 3), implies that these metabolites were transformed, and the biological activities of the enzymes POD and PPO, induced by silicon, could support it.^11^ These changes did not occur in the Si or Pc treatment, where the content of total (soluble) phenols was higher than that of the other treatments. There it could be inferred that the phenolic compounds were not modified into structures for the strengthening of the cell wall, allowing infection with the pathogen (Pc treatment).

For the SiPc treatment, which presented the highest activity of the PPO enzyme at 3 hpi, it could be inferred that it also presented the highest concentration of the product of the enzyme activity, that is, the quinones, which are known to reach be more toxic to the pathogen than the same phenols.^37^ It makes sense then that the Pc treatment was the one that presented the lowest activity of the PPO enzyme at the same time, thus avoiding the generation of antimicrobial compounds that could minimize the advance of *P. cinnamomi* in plants without an inducer.

Regarding the signaling involved in the expression of PPO, it seems that the participation of JA also plays a fundamental role when it corresponds to the defense response against a pathogen since this phytohormone is capable of positively regulating the expression of PPO^83^, [Jin^70^].

Although multiple studies suggest the induction of phenol production by silicon as a defense response,^14,15,84,85^ the analysis of the plant-pathogen interaction between Hass avocado and *P. cinnamomi*, allows us to state that these metabolites are not the main line of defense, at least directly, in Hass avocado plants elicited with silicon; PPO activity is proof of the above, however, it is necessary to evaluate the presence of antimicrobial compounds that could derive from the activity of the said enzyme, such as phytoalexins and/or quinones,^30^ to expand knowledge about the metabolism of phenolic compounds in the studied plant-pathogen interaction.

Finally, the high enzymatic activity of PPO in the SiPc treatment suggests that substrate availability must be present; however, the decrease in the content of phenols and the low activity of the PAL enzyme seem to show that the de novo synthesis of these metabolites was low in the defense response of Hass avocado under the conditions studied. Supporting the above,^86^,report that the beneficial effects of silicon become evident when plants are subjected to stress (biotic or abiotic), more than in those that grow under optimal conditions. All the above would strengthen the idea that the enzymatic activities of POD and PPO are the main responses related to the induction of defense with silicon in Hass avocado, as the maximum activity of such enzymes occurs in the SiPc treatment.

## 4. Conclusion

The action of the phenolic compounds was not direct during the defense response of Hass avocado elicited with silicon. That response depended mainly on the available enzymatic mechanism. A more detailed study on the composition of the phenols present in the evaluated samples would allow demonstrate the presence of these metabolites in more complex structures with a possible role in strengthening the cell wall. The possible deposition of silicon in plant tissue and the strengthening of the cell wall through the synthesis of compounds with a structural role, are proposed as the main defense tools during the interaction of Hass avocado with *P. cinnamomi*. Additionally, the action of the enzymes POD and PPO could have regulated the ROS and RNS, in addition to the generation of antimicrobial compounds as a product of the enzymatic activity induced by silicon. The main signaling pathway, suggested by the data obtained in SiPc treatment, corresponds to jasmonic acid signaling pathway, due in part to the low activity of the PAL enzyme, which is normally induced by salicylic acid; Additionally, jasmonic acid plays a fundamental role in the expression of POD and PPO enzymes. This research revealed fundamental aspects in the process of plant-pathogen interaction between Hass avocado plants in the greenhouse stage and the phytopathogen *Phytophthora cinnamomi*.
